# Propionic Acid Rescues High-Fat Diet Enhanced Immunopathology in Autoimmunity *via* Effects on Th17 Responses

**DOI:** 10.3389/fimmu.2021.701626

**Published:** 2021-06-01

**Authors:** Stefanie Haase, Jonas Mäurer, Alexander Duscha, De-Hyung Lee, Andras Balogh, Ralf Gold, Dominik N. Müller, Aiden Haghikia, Ralf A. Linker

**Affiliations:** ^1^ Department of Neurology, University Hospital Regensburg, Regensburg, Germany; ^2^ Department of Neurology, Friedrich-Alexander-University Erlangen-Nuremberg, Erlangen, Germany; ^3^ Department of Neurology, Otto-von-Guericke University, Magdeburg, Germany; ^4^ Experimental and Clinical Research Center, a Joint Cooperation of Max-Delbrück Center for Molecular Medicine and Charité-Universitätsmedizin Berlin, Berlin, Germany; ^5^ Department of Neurology, Ruhr University Bochum, Bochum, Germany; ^6^ Max-Delbrück Center for Molecular Medicine in the Helmholtz Association Berlin, Berlin, Germany; ^7^ German Center for Neurodegenerative Diseases (DZNE), Magdeburg, Germany

**Keywords:** high-fat diet, EAE, propionic acid, immunosuppression, multiple sclerosis

## Abstract

High-fat diets (HFD) are linked to obesity and associated comorbidities and induce pathogenic T helper (Th) 17 cells while decreasing regulatory T cells (Treg). This pro-inflammatory environment also aggravates immunopathology in experimental autoimmune encephalomyelitis (EAE) as a prototype model of T cell mediated autoimmunity. The strong association of HFD to obesity as well as the increasing risk of autoimmunity in the Western world stresses the importance to identify compounds that counteract this metabolically induced pro-inflammatory state in humans. One prominent candidate is the short-chain fatty acid propionate (PA) that was recently identified as potent therapy in the autoimmune disease multiple sclerosis by enhancing Treg cell frequencies and functionality. Mice were fed a HFD rich lauric acid (LA) and treated either with water or PA during MOG_35-55_-EAE. We analyzed Treg and Th17 cell frequencies in different tissues, antigen-specific cell proliferation and cytokine secretion, investigated Treg cell functionality by suppression assays and IL-10 signaling blockade and employed Western blotting to investigate the involvement of p38-MAPK signaling. Finally, we performed an explorative study in obese and non-obese MS patients, investigating fecal PA concentrations as well as peripheral Th17 and Treg frequencies before and after 90 days of daily PA intake. As compared to controls, mice on a HFD displayed a more severe course of EAE with enhanced demyelination and immune cell infiltration in the spinal cord. PA treatment prevented this disease enhancing effect of HFD by inhibiting Th17 mediated inflammatory processes in the gut and the spleen. Blocking experiments and signaling studies revealed p38-MAPK and IL-10 signaling as important targets linking the beneficial effects of PA treatment and reduced inflammation due to enhanced Treg frequency and functionality. An explorative study in a small group of MS patients revealed reduced PA concentrations in fecal samples of obese MS patients compared to the non-obese group, coinciding with increased Th17 but decreased Treg cells in obese patients. Importantly, PA intake could restore the Treg-Th17 homeostasis. Our data thus identify Th17 responses as an important target for the beneficial effects of PA in HFD and obesity in addition to the recently identified potential of PA as a Treg inducing therapy in T cell mediated autoimmunity.

## Introduction

An excess consumption of a so called ‘Western Diet’ has been linked to obesity, a long discussed risk factor for diabetes (1), hypertension (2), and arthritis (3). Moreover, observational studies demonstrated that obesity during young adulthood is associated with a higher risk of developing multiple sclerosis (4, 5), a T cell mediated neuroinflammatory disease. First murine studies showed that diet-induced obesity may impact regulatory T cells (Treg) and particularly promote T helper (Th) 17 driven immunity (6, 7), both cell types contributing to MS immunopathology. Later, we demonstrated that saturated long-chain fatty acids (LCFA) as integral components of the ‘Western Diet’ may link diet-induced obesity and multiple sclerosis risk (8). In the murine model of multiple sclerosis, experimental autoimmune encephalomyelitis (EAE), mice fed a LCFA-rich diet displayed a more severe disease course with an increased number of pro-inflammatory Th17 cells in the gut, the spleen and the spinal cord, while Treg numbers were decreased (8). This LCFA induced shift towards a pro-inflammatory environment may not only be relevant for EAE and multiple sclerosis, but also other diseases involving a T cell-mediated immunopathology such as rheumatoid arthritis, hypertension, atherosclerosis, psoriasis or inflammatory bowel disease (9–14). Moreover, the high prevalence of obesity and the number of related comorbidities makes it highly important to identify substances that counteract high-fat induced inflammation in T cell mediated diseases. As such, short-chain fatty acids (SCFA) may be of potential importance. SCFA such as acetate, butyrate or propionate (PA) are the main intestinal metabolites derived from gut microbiota processed dietary fiber. These compounds may regulate differentiation, activation and recruitment of immune cells. Besides local effects in the gut, SCFA may also harbor immunomodulatory capacities. Recently, we demonstrated that PA treatment decreases Th17 cell differentiation (8), while it increases Treg cells and enhances their suppressive capacity (8, 15, 16). The induction of an anti-inflammatory phenotype by SCFA treatment ameliorated clinical signs in EAE diseased mice with increased Treg numbers in the gut and spleen (8). These data from animal studies were transferred to humans where SCFA efficiently counteracted the progression of inflammatory diseases. Supplementation of PA to therapy-naive multiple sclerosis patients and as an add-on to multiple sclerosis immunotherapy significantly increased functionally competent Treg cells leading to reduced annual relapse rates together with reduced brain atrophy and a stabilization of disability (16). Another study in rheumatoid arthritis patients identified that high-fiber intake increased circulating Treg cells paralleled by improved disease-outcome (17), strengthening the potential of dietary SCFA supplementation to beneficially modulate immune-mediated diseases by the induction of Treg cells. We here used EAE as a prototype model of T cell immunopathology to investigate whether SCFA may counteract the high-fat diet-induced increase of Th17 cells by enhancing Treg cell responses, thereby preventing disease progression. These data could be translated to a small cohort of multiple sclerosis patients, indicating the importance for human diseases.

## Material and Methods

### Animal Experiments and Diet

All animal experiments were in accordance with the German laws for animal protection and were approved by the local ethic committees (Erlangen AZ 55.2 DMS-2532-2-27). C57Bl/6 mice (male and female) were housed in the animal care facility of the Friedrich-Alexander University Erlangen under a 12-h day-night cycle and standardized environmental conditions. Mice were fed control chow (ssniff EF R/M Control E15000-04, 4.2 % crude fat) or chow rich in the medium chain fatty acid lauric acid (LA; ssniff EF R/M E15116-34; 30.9 % crude fat) 4 weeks before EAE induction and had ad libitum access to food and water throughout the adaption and observation period. LA+PA mice received 150 mM propionic acid (PA) *via* oral gavage starting from the day of immunization in addition to LA-rich chow. Control and LA mice received water *via* oral gavage.

### EAE Induction

10-12 week-old mice were anaesthetized and subcutaneously injected with 200 µg myelin oligodendrocyte glycoprotein (MOG_35-55_) and 200 µg Complete Freund’s Adjuvant (CFA), containing 4 mg/ml *Mycobacterium tuberculosis* (H37RA). Mice received 200 ng pertussis toxin intraperitoneally on the day of immunization and two days later. Clinical scoring was performed on a 10-point scale (18).

### Histological Analysis

Mice were perfused with 4 % PFA on day 21 of EAE and spinal cord tissue was embedded in paraffin after 3 h post-fixation in 4 % PFA. 4 µm cross sections were deparaffinized in xylene and rehydrated in decreasing concentrations of ethanol before antigen retrieval in 1mM EDTA or citrate buffer. Antigens were blocked with 10 % BSA/PBS before incubation with the primary antibody over night at 4°C. Biotinylated secondary antibodies were applied for 60 min at room temperature followed by peroxidase-coupled avidin-biotin complex (ABC Kit, Vector Laboratories, Inc. Burlingame, CA). Reactivity was visualized with diamino-3,3’benzidine (DAB, Vector Laboratories, Inc. Burlingame, CA). Slides were counterstained using Mayer´s hemalaun solution (Merck, Darmstadt, Germany). The following antibodies were used: anti-CD3 (rat, 1:200, Biolegend), anti-Mac-3 (rat, 1:200, BD Biosciences), anti-GFAP (rabbit, 1:1000, Dako), anti-rabbit IgG (H+L) (goat, 1:200, Vector Laboratories), anti-rat IgG (H+L) (rabbit, 1:200, Vector Laboratories). Demyelination was assessed by luxol fast blue-periodic acid Schiff (LFB-PAS) staining. In brief, spinal cord cross sections were rehydrated in xylene and 96 % ethanol and incubated overnight in 0.1% LFB solution at 56°C. Sections were washed in 70% ethanol, incubated in 1% Li_2_CO_3_ for few seconds and transferred into 0.8% periodic acid solution for 10 min. Sections were stained in Schiff’s reagent for 20 min and washed in sulfite solution followed by further washing in *A.dest*., dehydrating and mounting. Axonal density was investigated with Bielschowsky silver staining. Rehydrated sections were incubated in 20% AgNO_3_ solution for 15 min at 37°C, washed with *A.dest.* and incubated in AgNO_3_ containing 25% NH_4_OH (AgOH solution) for 10 min in the dark at 37°C. After washing with 0.1% NH_4_OH solution, axons were visualized by adding developer stock solution (5% formaldehyde, one drop of 65% HNO_3_, 0,5g citric acid) to the AgOH solution for 4 min. Sections were washed with 0.1% NH_4_OH and *A.dest*. and fixed in 5% Na_2_S_2_O_3_ for 3 min followed by washing, dehydrating and mounting. Spinal cord cross sections were analyzed by a blinded observer using a BX-51 light microscope. Cellular infiltrates were counted in three lesions within each spinal cord segment (cervical, thoracic and lumbar) at 200x magnification within the margin of a stereological grid and quantified per square millimeter of white matter. Analysis of demyelinated areas in the white matter was performed semi-automatically with the help of CellP software. Axonal density was quantified in silver impregnated sections by counting on a 100 µm diameter grid.

### Isolation of Splenic Cells

Spleens were removed on day 10 of EAE and disrupted with a 5 ml glass homogenizer. Cells were filtered through a 100 µM cell strainer followed by erythrocyte lysis. Cells were washed with cold DPBS and used for flow cytometry analysis or the MOG_35-55_ restimulation assay.

### Isolation of CNS Infiltrating Cells

Spinal cord tissue was removed on day 21 of EAE after perfusion with cold DPBS and disrupted with a 5 ml glass homogenizer. Cells were transferred to a Percoll™ density gradient and centrifuged at 800g for 20 min without break. Cells at the interphases were collected, washed with cold DPBS and analyzed by flow cytometry.

### Isolation of Small Intestinal Lymphocytes

Single cell suspensions from lamina propria lymphocytes (LPL) were obtained from the small intestine on day 3 of EAE using the Lamina Propria Dissociation Kit (Miltenyi) following the manufacturer’s protocol. Isolated cells were purified with Percoll™ density gradient centrifugation, washed with cold DPBS and analyzed by flow cytometry.

### *In Vitro* MOG_35-55_ Restimulation Assay

Splenocytes were isolated on day 10 of EAE and seeded at a density of 3x10^6^ cells/cm^2^ in Re-medium. MOG_35-55_ (20 µg/ml) was added for stimulation and the cells were cultured 48 hours at 37°C. Supernatants were harvested and analyzed for secreted cytokines by ELISA. To monitor proliferation, cells were labelled with e450 proliferation dye (eBioscience) according to the manufacturer’s protocol. Proliferation was assessed 72h later by flow cytometry.

### Treg Suppression Assays

CD4+CD25+ cells were isolated from the spleen on day 10 of EAE using the CD4+CD25+ magnetic activated cell sorting (MACS) isolation kit (Miltenyi) according to the manufacturer’s instructions. CD4+CD25- cells from the same donor mice were stained with the e450 proliferation dye (eBioscience) according to the supplier’s protocol. Cells were seeded at different ratios (no Tregs, 1:2, 1:4, 1:8 and 1:16) on an anti-CD3 pre-coated (2 µg/ml, 145-2C11, BD Pharmingen) 96 well plate together with soluble anti-CD28 (2 µg/ml, 37.51, BD Pharmingen). Suppression capacity was analyzed 72h later by investigating proliferating CD4+CD25- cells *via* flow cytometry.

### Flow Cytometry Analysis

Dead cells were excluded by a fixable viability dye eFluor^®^780 (0.2 μl/test, eBioscience). Nonspecific Fc-mediated interactions were blocked by addition of 0.5 μl anti-CD16/32 (93, eBioscience) for 10 min. For surface staining, cells were stained with the respective fluorochrome conjugated antibodies for 30 min in PBS. For intracellular cytokine staining, cells were stimulated for 4 h with ionomycin (1 µM) and PMA (50 ng/ml) in the presence of monensin (2 μM). Cells were stained for surface marker and made permeable by saponin buffer or Fix/Perm (eBioscience) according to the manufacturer’s protocol. Intracellular cytokines were stained with the respective fluorochrome conjugated antibodies for 30-45 min. The following antibodies were used: CD4 (RM4-5, eBioscience), CD25 (PC61, BioLegend), FoxP3 (FJK-16s, eBiosciences), IL-17A (eBio17B7, eBioscience), IL-10 (JESS-16E3, BioLegend), IFN-γ (XMG1.2, BD Biosciences).

### Western Blot Analysis

Small intestinal LPL were isolated from control, LA and LA+PA mice on day 3 of EAE. Splenic cells were isolated on day 10 of EAE. Cells were treated with 1x Ripa lysis buffer [10xRipa: 150 mM NaCl, 38.5mM SDS, 50mM Tris, 134mM SDOX, 0.5 mM EDTA, 1% NP40, complete protease inhibitor cocktail, Complete Mini and phosphatase inhibitor cocktail, PhosStop (Roche Diagnostic GmbH, Mannheim, Germany)] and centrifuged for 10 min at 10,000rpm. Protein concentration was determined with the BC Assay Protein Quantitation Kit (Interchim). p38 protein was detected by using rabbit anti-p38 (Cell Signaling Technology, 1:500). Phosphorylation of p38 protein was detected by using rabbit anti-phospho-p38 (Cell Signaling Technology, 1:500). Mouse anti-β-actin (1:1000) was obtained from Millipore (clone C4). C-6 glioma cells with or without anisomycin treatment were used as pp38 positive or negative control (Cell Signaling Technology). Goat anti-mouse Alexa Fluor 488 conjugated and goat anti-rabbit Alexa Fluor 647 conjugated secondary antibodies (1:1000, Invitrogen) were used. Detection was performed with the Fusion Capt Advance FX7.

### Cytokine Measurement

Cytokine concentrations in cell culture supernatants were analyzed by enzyme linked immunosorbent assay (ELISA) for the secretion of IL-17A, IL-10 and IFN-γ (ELISA DuoSet, R&D) according to the manufacturer’s protocol.

### *In Vitro* T Cell Differentiation Assay

Spleens from naïve C57Bl/6 mice were digested for 30 min in RPMI media containing DNaseI (10 mg/ml) and Liberase TL (1.67 Wünsch Units/ml). T cells were isolated by MACS using the Pan T cell isolation kit (Miltenyi). Cells were fluorescently stained with anti-CD4-FITC (RM4-5, eBioscience), anti-C25-PECy5 (PC61.5, eBioscience), anti-CD44-PE (IM7, BioLegend) and anti-CD62L-APC (MEL-14, eBioscience) to isolate naïve T cells with a cell sorter. Naïve T cells were cultured on 96 well plates together with plate-bound anti-CD3 (2 µg/ml, 145-2C11, BD Pharmingen) and anti-CD28 (2 µg/ml, 37.51, BD Pharmingen) and rhTGF-β1 (1 ng/ml) for Treg cell differentiation. The cells were additionally treated with solvent (PBS), LA (250µM) or LA+PA (PA 150µM) for 4 days and analyzed for the frequency of CD25+FoxP3+ cells in CD4+ viable lymphocytes with flow cytometry. In some experiments, cells were additionally treated with an IL-10 receptor blocker (1µg/ml, Biolegend).

### Human Study

The human study was approved by the ethics committee of the Department of Medicine at the Ruhr-University Bochum (registration number 15-5351, 4493-12, 17-6235). Prior to study participation, all participants signed informed consent forms. We performed an explorative subgroup analysis from a previously published large cohort of PA-treated RRMS patients (16) and analyzed the MS patients for body height and weight to determine their age dependent body-mass index (BMI). A BMI ≥ 30 defined MS patients as obese **(** https://www.cdc.gov/obesity/adult/defining.html), non-obese MS patients had a BMI up to 30. All MS patients were instructed to supplement 500 mg sodium-propionate (PA) capsules twice daily for 90 days (Flexopharm, Herne, Germany).

### Flow Cytometry Analysis in Human Blood

Blood samples were analyzed before and after termination of PA intake. Th17 cells were identified with αCD196 (CCR6)-PerCP Cy5.5 (11A9; BD), αCD4-FITC (RPA-T4; BD), αCD161-PE (DX-12; BD), αCD183 (CXCR3)-APC (1C6/CXCR3; BD) and IL-17 (BD Bioscience) as previously described (16). For Treg analysis, cells were stained with αCD4-FITC (RPA-T4, eBioscience), αCD25-APC (BC96, eBioscience) and intracellular αFoxP3-PE (236A/E7, eBioscience) by using Foxp3/Transcription Factor Staining Buffer Set (eBioscience) according to manufacturer protocol. Phenotyping was performed on BD FACS Canto2 and analyzed by BD FACS DIVA v6 software.

### Quantification of PA in Feces

PA in fecal samples was analyzed as described previously  (16, 19). In brief, 10 mg of fecal samples were homogenized with HCl, ether and crotonic acid as internal standard. The ether layer was mixed with N-tert-butyldimethylsilyl-N-methyltrifluoroacetamide (MTBSTFA), derivatized at room temperature for 24h and analyzed by high-performance liquid chromatography-tandem mass spectrometry (LC-MS/MS).

### Statistical Analysis

Statistical analysis was performed using Graph Pad Prism. All murine *in vitro* and *ex vivo* data were analyzed by one- or two-way ANOVA followed by Tukey`s posttest. EAE data were analyzed by Kruskal-Wallis test. Human data were analyzed by two-tailed Mann-Whitney test. Data are presented as mean ± SEM; *p<0.05, **p<0.01, or ***p<0.001 were considered to be statistically significant.

## Results

### PA Rescues the LA-Diet Enhanced Immunopathology in MOG_35-55_-EAE by Increasing Treg Cell Frequencies

**Figure 1:**
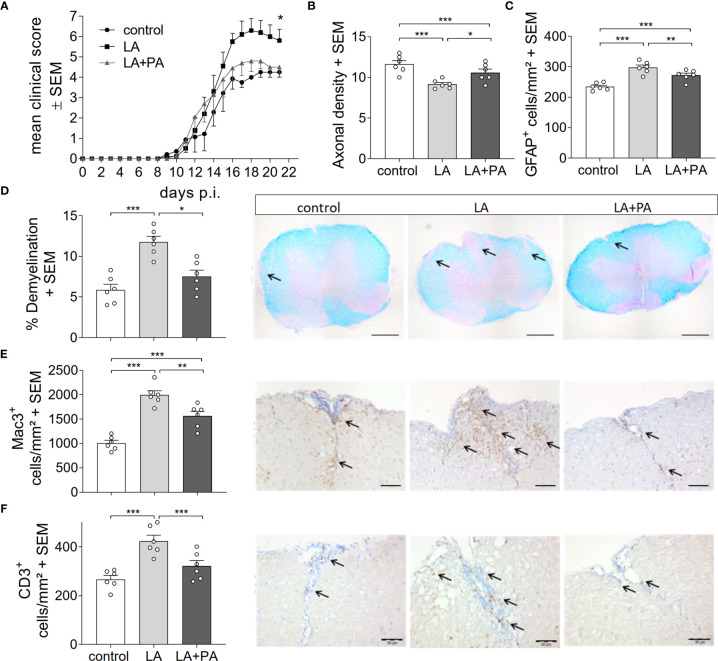
PA reverts the deleterious effect of a LA-rich diet in MOG-EAE. **(A)** Clinical course of MOG_35-55_-EAE. Mice were fed a LA-rich diet or control-diet 4 weeks prior to immunization and were additionally treated with PA (LA+PA, 150mM) or water (control; LA) by daily oral gavage starting from day 0. Data are shown on a 10-point score scale as mean ± SEM and are pooled from two independent experiments (control n=11, LA n=11, LA+PA n=12). **(B–F)** Histological analysis of spinal cord cross sections on day 21 of EAE. **(B)** Axonal density was analyzed by Bielschowsky silver staining. **(C)** Analysis of GFAP+ astrocytes. **(D)** Demyelination was analyzed by LFB-PAS staining. **(E)** Infiltration of macrophages (Mac-3) and **(F)** CD3 positive T cells are reduced in LA-diet mice treated with PA compared to control treated animals. Representative pictures are shown for each staining and group. Scale bars are 500 µm for LFB-PAS and 50 µm for Mac-3 and CD3. Arrows indicate CD3+ cells, Mac3+ cells or demyelinated area. n=6 mice per group. *p<0.05 using Kruskal-Wallis test for **(A)**, *p<0.05, **p<0.01, ***p<0.001 using one-way ANOVA and Tukey’s posthoc test for **(B–D)**. p.i., post immunization; LA, lauric-acid diet; PA, propionic acid.

**Figure 2:**
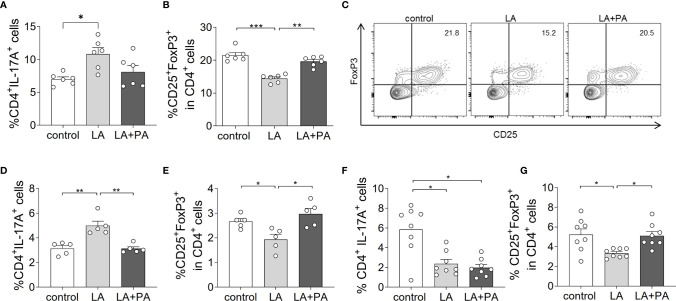
PA treatment reverts the pro-inflammatory effect of a LA-rich diet and increases Treg cell frequencies during MOG-EAE. Mice were fed a LA-rich diet or control-diet 4 weeks prior to immunization and were additionally treated with PA (LA+PA) or water (control; LA) by daily oral gavage starting from day 0. **(A–C)** *Ex vivo* flow cytometry analysis of **(A)** CD4+IL-17+ Th17 cells and **(B)** CD25+FoxP3+ Treg cells in the spinal cord on day 21 of EAE (n=6 per group). **(C)** Representative flow cytometry images of CD25+FoxP3+ Treg cells in the spinal cord on day 21 of EAE. **(D, E)** *Ex vivo* flow cytometry analysis of **(D)** CD4+IL-17+ Th17 cells and **(E)** CD25+FoxP3+ Treg cells in the spleen on day 10 of EAE (n=5 per group). **(F, G)** *Ex vivo* flow cytometry analysis of **(F)** CD4+IL-17+ Th17 cells and **(G)** CD25+FoxP3+ Treg cells in the small intestine on day 3 of EAE (n=8 per group). *p<0.05, **p<0.01, ***p<0.001 using one-way ANOVA and Tukey’s posthoc test.

To investigate whether PA can revert the deleterious effects of long-chain fatty acids during neuroinflammation, we fed mice a standardized diet rich in the C12:0 fatty acid LA 4 weeks prior to the induction of MOG_35-55_-EAE and treated mice either with water (LA) or PA (LA+PA) starting from the day of immunization. A control group was fed a matched control diet and was treated with water from the day of immunization. All mice had comparable body weights prior to EAE induction independent of the diet (control: 20.8 ± 0.6 g versus LA: 21.4 ± 1.3 g, p=0.679). Clinical scoring revealed a more severe EAE course in LA-diet mice compared to the control diet group and treatment with PA in LA-diet fed mice resulted in an amelioration of clinical signs (**Figure 1A**). The disease course of LA-diet mice treated with PA was comparable to that of mice receiving a control diet. Disease incidence (control: 10/11, LA: 10/11, LA+PA: 11/12) and mortality were neither affected by LA-diet feeding nor by PA treatment. The improved clinical outcome in LA-diet mice treated with PA was reflected in the histopathological analyses of the spinal cord on day 21 of EAE (**Figures 1B–F**). LA-diet mice treated with PA showed a significantly enhanced axonal density (**Figure 1B**), reduced astrocyte numbers (**Figure 1C**) and reduced demyelination (**Figure 1D**) compared to control-treated LA-diet mice. Moreover, LA-diet feeding resulted in increased numbers of infiltrating macrophages (**Figure 1E**) and T cells (**Figure 1F**) compared to the controls, while treatment with PA in LA-diet fed mice significantly reduced immune cell infiltration in the spinal cord. Additional histological analyses revealed no effect on Olig2+ cells and neurons (data not shown). We already showed that LA induces Th17 cells during MOG-EAE while PA increases Treg cell frequencies and their suppressive capacity (8). Therefore, we additionally performed *ex vivo* flow cytometry analysis of CD4+IL-17A+ Th17 and CD4+CD25+Foxp3+ Treg cells in the spinal cord on day 21 of EAE. We observed increased Th17 cell frequencies in LA-diet fed mice compared to control-diet mice, whereas LA-diet fed mice treated with PA showed a trend towards reduced Th17 frequencies nearly to the control level (**Figure 2A**). In contrast, mice fed a LA-diet showed decreased Treg frequencies, while PA treatment significantly increased Treg cells, comparable to the Treg frequencies observed in the control group (**Figures 2B, C**). These effects were also observed in the spleen prior to the onset of EAE symptoms. *Ex vivo* flow cytometry analysis on day 10 of EAE revealed decreased Th17 cell frequencies in PA-treated LA-diet mice compared to control-treated LA-diet mice (**Figure 2D**). In contrast, PA treatment restored Treg cell frequencies in LA-diet mice to a level observed in the control group (**Figure 2E**). We next investigated the gut as a prominent anatomic site of fatty acid interaction with T cells (8). *Ex vivo* flow cytometry analysis of the small intestine on day 3 of EAE showed a decrease of Th17 cell frequencies in LA-diet mice compared to the controls independent of PA treatment (**Figure 2F**). However, PA treatment in LA-diet mice increased Treg cell frequencies in the gut, thus inhibiting the LA-diet induced decrease of Treg cells compared to controls (**Figure 2G**). In contrast, Th1 cells were not altered in the spinal cord, the spleen or the gut by LA-diet feeding and PA treatment (data not shown). Our data thus indicate that PA treatment rescued the disease enhancing effects of a LA-diet by inhibiting Th17 cell expansion and increasing Treg cell frequencies.

### PA Reverts the Deleterious Effect of a LA-Rich Diet by Increased IL-10 Production and Enhanced Suppression Capacity of Treg Cells

**Figure 3:**
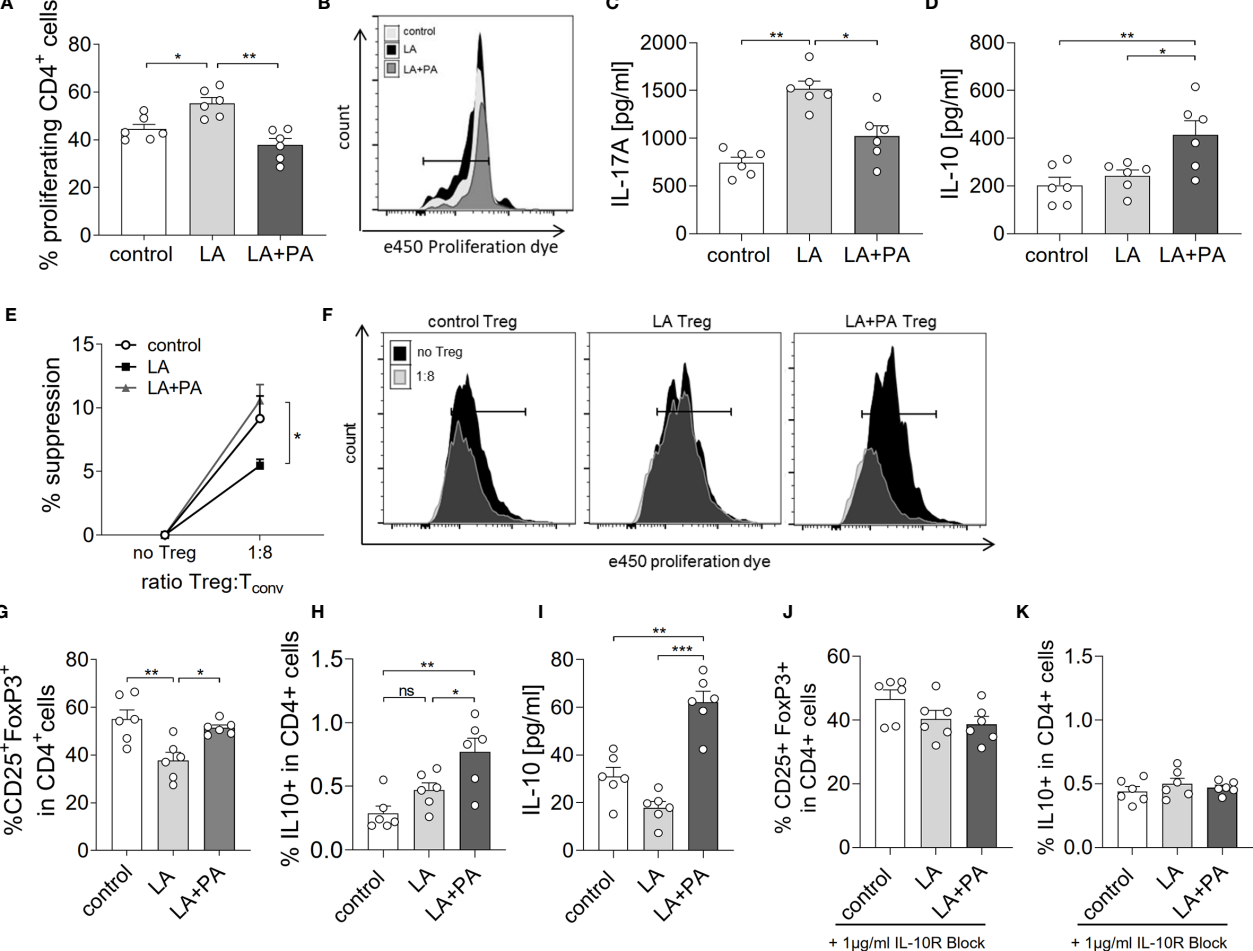
PA reverts the deleterious effect of a LA-rich diet by increased IL-10 production and enhanced suppression capacity of Treg cells. Mice were fed a LA-rich diet or control-diet 4 weeks prior to immunization and were additionally treated with PA (LA+PA) or water (control; LA) by daily oral gavage starting from day 0. **(A, B)** *Ex vivo* proliferation assay of CD4+ T cells isolated from the spleen on day 10 of EAE. Isolated cells were re-stimulated with MOG_35-55_ *in vitro* and proliferation was assessed by flow cytometry 72h later. **(A)** Quantification of proliferating cells from control, LA or LA+PA treated mice (n=6 per group). **(B)** Representative histograms of proliferating CD4+ T cells. **(C, D)** Splenic cells were isolated on day 10 of EAE and re-stimulated with MOG_35-55_ *in vitro* for 48h. Quantification of **(C)** IL-17A and **(D)** IL-10 in cell culture supernatants analyzed by ELISA (n=6 per group). **(E, F)** Treg suppression assay of *ex vivo* obtained CD4+CD25+ splenic T cells isolated from control, LA or LA+PA treated mice on day 10 of EAE. CD4+CD25+ T cells were co-cultured with e450 labeled CD4+CD25- cells for 72h (n=5 per group). **(F)** Representative histograms of proliferating CD4+ cells co-cultured with or without Treg cells analyzed by flow cytometry. **(G–I)** Splenic naïve CD4+ T cells were treated with PBS (control), LA (250µM) or LA+PA (PA 150µM) under Treg polarizing conditions. Flow cytometry analysis of CD25+FoxP3+ cells **(G)** and IL10+FoxP3+ cells **(H)** in viable CD4+ cells was performed 96h later (n=6 from 2 independent experiments). **(I)** Quantification of IL-10 in cell culture supernatants of Treg differentiation assays analyzed by ELISA. **(J, K)** IL-10 signaling was blocked by the addition of an IL-10 receptor blocker (1µg/ml) to CD4+ T cell differentiation assay under Treg polarizing conditions (n=6 from two independent experiments). Flow cytometry analysis of CD25+FoxP3+ cells **(J)** and IL10+FoxP3+ cells **(K)** in viable CD4+ cells was performed 96h later (n=6 from 2 independent experiments). ns, not significant. *p<0.05, **p<0.01, ***p<0.001 using one-way ANOVA and Tukey’s posthoc test.

**Figure 4:**
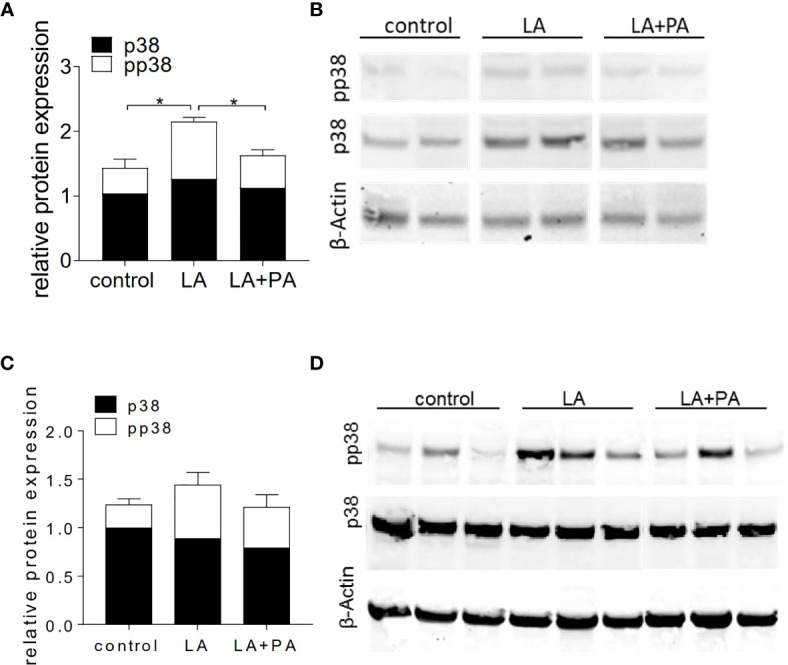
PA decreases the LA-induced phosphorylation of p38-MAPK in the spleen and the small intestine. Mice were fed a LA-rich diet or control-diet 4 weeks prior to immunization and were additionally treated with PA (LA+PA) or water (control; LA) by daily oral gavage starting from day 0. **(A, B)** Western Blot analysis of p38 and phosphorylated p38 (pp38) from small intestinal LPL isolated on day 3 of EAE. **(A)** Quantification of p38 expression (black) and pp38 (white) in control, LA and LA+PA treated mice normalized to β-Actin expression. **(B)** Representative Western Blot images. **(C, D)** Western Blot analysis of p38 and pp38 from splenocytes isolated in day 10 of EAE. **(C)** Quantification of p38 expression (black) and pp38 (white) in control, LA and LA+PA treated mice normalized to β-Actin expression. **(D)** Representative Western Blot images. *p<0.05 using one-way ANOVA and Tukey’s posthoc test.

We next investigated the potential mechanism of PA to reduce the LA-diet enhanced immunopathology during neuroinflammation. *Ex vivo* analysis of the spleen in a recall assay with MOG_35-55_ revealed an increased proliferation of CD4+ T cells in splenocytes from LA-diet mice compared to the control group (**Figures 3A, B**). Yet, PA-treatment during LA-diet feeding blunted the enhanced proliferation of CD4+ T cells and reduced proliferating T cell frequencies to levels observed in the control-diet group. Moreover, *ex vivo* recall assays revealed a significantly enhanced IL-17A secretion in splenocytes isolated from LA-diet mice compared to the controls, which was decreased by PA treatment during LA-diet feeding (**Figure 3C**). Interestingly, we observed a significant increase of the anti-inflammatory cytokine IL-10 in cells derived from LA-diet mice treated with PA compared to the control-treated LA-diet group and mice receiving a control diet (**Figure 3D**). Since IL-10 acts as a suppressor cytokine of enhanced pro-inflammatory immune cell responses, we next investigated the suppressive capacity of Treg cells isolated from control mice, control-treated LA-diet mice and LA-diet mice treated with PA (**Figures 3E, F**). The suppression of CD4+ effector T cells was significantly reduced by Treg cells isolated from LA-diet fed mice (**Figure 3E**, black squares). In contrast, PA treatment during LA-diet feeding normalized the suppressive capacity of Treg cells (**Figure 3E**, grey triangle) to a level observed in the control group (**Figure 3E**, white dots). We next performed *in vitro* T cell differentiation assays to assess the potential link of PA treatment and IL-10 secretion in Treg cells. The addition of PA to T cell cultures treated with LA normalized Treg cell frequencies to comparable levels observed in controls (**Figure 3G**). Moreover, PA treatment significantly increased the frequencies of IL-10 producing Treg cells as detected by flow cytometry (**Figure 3H**) and ELISA (**Figure 3I**). We next blocked the IL-10 signaling pathway during Treg differentiation by adding an IL-10 receptor blocker (IL-10R Block, 1µg/ml). This blockade inhibited the effect of PA on Treg cell differentiation, as we observed similar FoxP3+ Treg cells (**Figure 3J**) and IL-10 producing Treg cells (**Figure 3K**) in all investigated groups. Our data thus indicate that PA enhances the IL-10 secretion in Treg cells, thereby boosting the suppressive capacity of Treg cells. We further investigated the potential involvement of the mitogen-activated protein kinase (MAPK) signaling pathway p38 as relevant contributor to enhanced Th17 induction by LA (8). At the post-transcriptional level, PA treatment in LA-diet fed mice inhibited the increase of p38 phosphorylation as observed in control-treated LA-diet mice (**Figure 4**). This effect was even more pronounced in the small intestine on day 3 of EAE (**Figure 4A**) compared to the spleen analyzed prior to the onset of EAE symptoms (day 10 p.i., **Figure 4B**). These data indicate that PA reverts the deleterious effect of a LA-rich diet by decreasing the LA-induced phosphorylation of p38-MAPK.

### PA Supplementation in Obese MS Patients Can Restore the Imbalanced Th17-Treg Homeostasis

**Figure 5:**
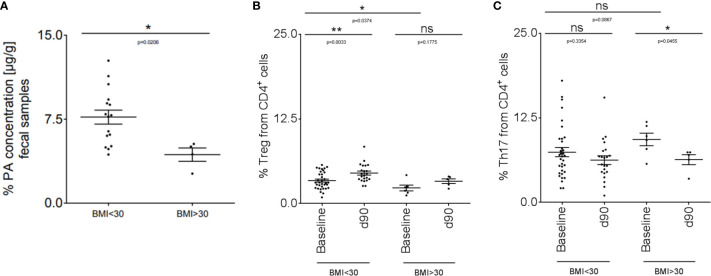
PA supplementation in obese MS patients can restore the imbalanced Th17-Treg homeostasis. **(A)** PA concentrations were analyzed in fecal samples of non-obese (BMI <30; n=16) and obese (BMI >30; n=4) MS patients by LC-MS/MS. **(B, C)** Flow cytometry analysis of **(B)** Treg and **(C)** Th17 cells in the blood of non-obese (BMI <30) and obese (BMI >30) MS patients at baseline and after 90 days of PA intake (1000mg per day). BMI <30: baseline n=34, d90 n=22. BMI >30: baseline n=6, d90 n=5. p values are indicated in the figure and were determined by Mann-Whitney test. ns, not significant. *p<0.05, **p<0.01 using Mann-Whitney test.

We translated our findings to human disease and performed an explorative study in a small cohort of MS patients (disease characteristics are summarized in **Table 1**). We included MS patients with a body mass index (BMI) higher than 30 (obese MS patients) compared to MS patients with a BMI below 30 (non-obese). We first investigated fecal PA concentrations in both groups to prove whether obesity might further affect the altered gut microbiota metabolomics of MS patients as observed previously  (16). Compared to non-obese MS patients, fecal PA concentrations were significantly reduced in obese MS patients (**Figure 5A**). We next hypothesized that reduced PA concentrations in the gut may coincide with altered immune cell frequencies in obese MS patients that may be restored by PA supplementation as observed in our murine data. To investigate this, we analyzed Th17 and Treg cell frequencies in the blood at baseline and after 90 days of PA supplementation as add-on therapy to the pre-existing immunomodulatory therapy. At baseline, obese MS patients displayed reduced Treg frequencies (**Figure 5B**) but increased Th17 frequencies (**Figure 5C**) compared to the non-obese group, indicating an obesity-related imbalance of Th17 and Treg cells. Importantly, PA supplementation increased Treg frequencies in both MS-groups, albeit only statistically significant in non-obese MS patients (**Figure 5B**). Moreover, Th17 cell frequencies were significantly decreased in obese MS patients after PA intake, and showed a trend towards lower cell counts in non-obese MS patients supplemented with PA compared to the baseline (**Figure 5C**). Our data thus indicate that PA supplementation in MS patients is able to restore the obesity-associated imbalance of Th17-Treg homeostasis, probably involving an alteration of fecal PA concentrations.

**Table 1:** MS patient characteristics.

Characteristics	MS BMI<30	MS BMI>30
	(n=34)	(n=6)
Female sex, n (%)	19 (55.8%)	3 (50%)
Age, years, mean ± SD	55,70 ( ± 10.54)	56,14 ( ± 11.63)
Subgroups		
RRMS	9 (26.5%)	2 (33.3%)
SPMS	20 (58.8%)	2 (33.3%)
PPMS	5 (14.7%)	2 (33.3%)
Therapy regimen		
Interferon B	2	0
Dimethylfumarate	7	2
Fingolimod	1	0
Steroids ithc.	26	5

## Discussion

Our data add to the recently identified property of LCFA to exacerbate EAE by shifting the T cell pool towards pro-inflammatory Th17 cells (8). We observed a LCFA-induced increase of Th17 cells in the spinal cord as target organ during EAE, as well as increased Th17 frequencies in the spleen and the gut prior to the onset of EAE symptoms. More importantly, we describe the potential of dietary SCFA supplementation to counteract this disease enhancing effect by inducing functionally competent Treg cells that prevent the Th17-mediated exacerbation *via* increased IL-10 secretion. We translated these findings to a small cohort of MS patients and identified an imbalanced Th17-Treg homeostasis in obese patients paralleled by reduced PA concentrations in the gut. Interestingly, PA supplementation could restore the Th17/Treg balance in MS patients, demonstrating the relevance of our findings for human diseases involving a T cell-centered immunopathology.

PA treatment in mice fed a LCFA-diet restored Treg cell frequencies in the spinal cord, together with the prevention of LA-induced EAE severity. This Treg cell induction was initiated in the small intestine prior to the onset of EAE symptoms, identifying the gut as a prominent compartment mediating the preventive effect of PA treatment on LCFA-enhanced immunopathology in neuroinflammation. Indeed, the gut is the primary absorption interface for nutrients and an important tolerogenic environment shaping our immune system (20–22). Moreover, the gut commensal bacteria are highly important contributors to EAE pathology (23, 24) and different bacterial species are implied in either Th17 or Treg cell development (25–27). Vice versa, IL-17 secreting Th17 cells were recently shown to modulate the intestinal microbiota, thereby indirectly affecting CNS autoimmunity (28). Albeit these data firstly question the encephalitogenicity of IL-17 during EAE, they support the importance of the gut and the gut microbiota as relevant Th17-affecting compartment during neuroinflammation. Moreover, the gut microbiota composition is mainly affected by antibiotic intake (29) and dietary habits (30), and we recently observed that diets rich in LCFA alter the gut microbiota composition towards a Th17 cell inducing environment (8). LCFA intake decreases SCFA concentrations in the gut, probably reducing Treg cell functionality during EAE (8). The here presented beneficial effect of PA treatment on the high-fat diet-enhanced immunopathology may therefore be linked to (i) a prevention of the LCFA-induced alteration of the gut microbiota composition towards Th17 inducing microorganisms, (ii) restoration of microbial species inducing an anti-inflammatory environment, or (iii) a short-term increase of mucosal PA concentrations with direct consequences on Treg cells. Particularly the first two points need to be proven in future studies, investigating the gut microbiota composition in LCFA-diet fed mice treated with PA or vehicle.

PA treatment during LCFA-intake could restore the suppressive capacity of Treg cells during EAE, correlating with enhanced IL-10 secretion as detected in an antigen-specific re-stimulation assay and in *in vitro* Treg cultures. Of note, IL-10 signaling in Treg cells is required for the suppression of Th17 cell-mediated inflammation (31), strengthening our concept that PA rescues the LCFA enhanced immunopathology by increasing the IL-10 mediated suppressive capacity of Treg cells. Yet, we cannot necessarily exclude that other mechanisms besides IL-10 secretion might be responsible for the increased Treg suppressive capacity *in vivo*. As such, suppression by modulation of dendritic cell maturation or metabolic disruption may be of potential importance (32, 33). However, Treg cell-derived IL-10 seems to be required for the control of inflammatory events at mucosal interfaces including the lungs and the colon (34), supporting our hypothesis that PA prevents a Th17-mediated immunopathology involving the gut. While other studies identified a potential involvement of STAT3 phosphorylation in IL-10 mediated suppression of Treg cells (35), we propose the involvement of the p38 MAPK signaling pathway, a well-known integrator of environmental stress that has previously been shown to be involved in T cell differentiation (36) and to be critical in models of MS (37). Our data confirm previous studies, demonstrating that LA treatment increases p38 phosphorylation in Th17 cells, whereas Treg cell differentiation correlated with decreased p38-MAPK activation (8).

The here presented data are not only relevant for EAE as a prototype model of T cell-mediated neuroinflammation, but also for other diseases involving an imbalance in Th17-Treg homeostasis. Moreover, LCFA such as LA are a major component of our modern ‘Western diet’. Hence, our data may be of potential importance when investigating dietary induced obesity. This hypothesis is strengthened by our explorative study with a small group of PA-treated MS patients, demonstrating that obesity in T cell mediated neuroinflammation affects the fecal metabolite composition and shifts the T cell balance towards increased pro-inflammatory T cells. The observed reduction of fecal PA concentrations in obese MS patients compared with non-obese patients might be related to an altered microbiota composition and functionality as recently indicated (38). However, analysis of the gut microbiome composition and larger sample sizes will be necessary to prove this concept. Yet, our data add to the observation that obesity correlates with an imbalance in peripheral Th17-Treg homeostasis (39). We observed altered Treg and Th17 cell numbers in MS patients with a BMI higher than 30 compared to normal-weight patients. Until now, only *in vitro* studies identified a potential benefit of PA on obesity-associated inflammation (40, 41). To our knowledge, this is the first study demonstrating that PA supplementation may counteract obesity-enhanced pro-inflammatory immune responses in neuroinflammatory diseases, further attracting its therapeutic potential. Yet, we performed an explorative study in a small number of obese MS patients and larger cohorts will be necessary in the future to confirm the therapeutic potential of PA treatment in obese patients. Moreover, we solely analyzed immune cell frequencies in the blood rather than investigating a direct link of PA intake and the clinical outcome of obese MS patients. However, we could recently demonstrate that PA supplementation to MS patients beneficially affected the annual relapse rates and disease stability due to increased Treg cells (16), indicating that the observed restoration of Treg cells in obese MS patients may also improve their clinical outcome. It is long accepted that obesity correlates with metabolic alterations and systemic inflammation, contributing to severe comorbidities such as type 2 diabetes, cardiovascular diseases, asthma and certain types of cancer (42–45). Therefore, the here identified benefit of PA supplementation in obese people may also prevent obesity associated comorbidities, making its therapeutic potential even more attractive.

## Data Availability Statement

The original contributions presented in the study are included in the article/supplementary material. Further inquiries can be directed to the corresponding author.

## Ethics Statement

The studies involving human participants were reviewed and approved by Ethics committee of the Department of Medicine at the Ruhr-University Bochum (registration number 15-5351, 4493-12, 17-6235). The patients/participants provided their written informed consent to participate in this study. The animal study was reviewed and approved by Local ethic committees Erlangen AZ 55.2 DMS-2532-2-27.

## Author Contributions

SH designed and conceptualized the study, acquired and analyzed the data and wrote the manuscript. JM and D-HL acquired and analyzed data. AD and AB acquired and analyzed human data. RG, DM and AH revised the manuscript for intellectual content. RL designed and conceptualized the study and revised the manuscript for intellectual content. All authors contributed to the article and approved the submitted version.

## Funding

The study was funded by SFB TR128 (Deutsche Forschungsgemeinschaft), the DMSG (Deutsche MS Gesellschaft in NRW), FoRUM (Ruhr-University Bochum), the Rose-Stiftung, ProDi (Proteindiagnostik RUB), the Ministerium f̈r Kultur und Wissenschaft des Landes Nordrhein-Westfalen, the Regierende B̈rgermeister von Berlin – inkl. Wissenschaft und Forschung, and the Bundesministerium f̈r Bildung und Forschung. DM was supported by the Deutsche Forschungsgemeinschaft (DFG, German Research Foundation; Projektnummer 394046635 - SFB 1365) and by the DZHK (German Center for Cardiovascular Research, 81Z0100106).

## Conflict of Interest

The authors declare that the research was conducted in the absence of any commercial or financial relationships that could be construed as a potential conflict of interest.
